# Food safety and hygiene education improves the knowledge, attitudes, and practices of Saudi dietetics students

**DOI:** 10.6026/97320630018900

**Published:** 2022-10-31

**Authors:** Wejdan T Alghafari, Leila Arfaoui

**Affiliations:** 1 Clinical Nutrition Department, Faculty of Applied Medical Sciences, King Abdulaziz University, Jeddah, Saudi Arabia

**Keywords:** Dietetics students, knowledge, attitude, practice, food safety and hygiene course, education

## Abstract

The objective of the study is to evaluate the effect of food safety and hygiene course on the knowledge, attitudes, and practices of dietetics students. A repeated measure pre/post-intervention study was used to assess the knowledge, attitudes, and self-reported practices regarding food safety and hygiene among undergraduate dietetics students (n = 63) enrolled in a course on food safety and hygiene in Saudi Arabia. Students were asked to complete an online questionnaire divided into five sections that covered key food-safety concepts before and at the end of the course to determine changes in these variables. Overall, students’ knowledge, attitudes, and practices improved significantly after attending the course on food safety and hygiene. Scores for total knowledge increased from 16.51±2.60 before the course to 20.60±2.01 after it (p < 0.001). The total score for attitudes improved from 9.16±1.43 before the course to 9.92±0.27 after it (p < 0.001). The total score for practices increased from 11.0±3.10 before the course to 14.78±3.41 after it (p < 0.001).The course on food safety and hygiene helped to improve the knowledge, attitudes, and self-reported practices of food safety by dietetics students.

## Background:

Food safety is an important aspect of community health and disease prevention [1]. Dietitians play an important part in educating the public on how to eat a balanced and healthy diet and, most importantly, how to prepare food safely to avoid the risk of foodborne illnesses [2]. Moreover, dietitians working in hospitals have a fundamental role in providing safe food for patients and counselling patients who are highly susceptible to foodborne illnesses, including pregnant women, the elderly, or immunocompromised patients [2] . Studies have revealed the public consider dietitians to be health professionals that they trust to provide advice on food safety [3]. Medeiros et al. reported that immunocompromised patients trust dietitians to provide information on low-bacteria diets prescribed to reduce the risk of foodborne illnesses [4]. As professionals of the future, dietetics students must be given an appropriate depth and quality of information to provide effective education on food safety. The international curricula for the training of undergraduate dietitians include a food-safety course [5, 6]. Furthermore, the Accreditation Council for Education in Nutrition and Dietetics accreditation standard for the Didactic Program in Dietetics (DPD) has listed food science as a required component of the curriculum for dietetics students. In addition, under "Standard 5: Core Knowledge for the RDN (KRDN)," upon completion of the DPD, graduates will be able to "Describe safety principles related to food, personnel and consumers". Also, under "Standard 5: competencies for the RDN (CRDN)", upon completion of the DPD, graduates will be able to "undertake the management functions related to safety, security and sanitation that affect employees, customers, patients, facilities and food" [7]. Therefore, the current program on clinical nutrition at King Abdulaziz University (Jeddah, Saudi Arabia) provides a course on food safety and hygiene as a required curriculum component to second-year students at the bachelor-degree level. This course is designed to equip undergraduate dietetics students with the knowledge and skills required to prepare, handle, and store food safely, to understand the practices of personal hygiene, and to recognize the factors commonly associated with foodborne illnesses.

Several studies have determined the level of knowledge, attitudes, and practices of food safety for university students from different backgrounds. Scholars found that students had a relatively low level of knowledge of food safety [8-11]. Studies assessing the level of knowledge of food safety, attitudes, and practices among students of Taif University (At Taif, Saudi Arabia) and King Saudi University (Riyadh, Saudi Arabia) reported that students were fairly knowledgeable of foodborne illnesses, and that most of them considered health professionals or government Internet websites to be their main source of information [12-14]. In addition, studies have shown that students studying at food- or health-related faculties (e.g., dietetics students) [11,15] or who had passed food-related courses had a much higher level of knowledge and attitudes of food safety than non-health-related students [16,17]. A study in the USA reported the effectiveness of an educational intervention on food safety among university students. That study showed that an educational intervention improved the attitudes, beliefs, and knowledge of food safety of college students, with the strongest effects seen in students majoring in health compared with those not majoring in health [18]. A study to evaluate the effectiveness of an educational intervention of food safety among dietetics students has not been conducted. We evaluated the knowledge, attitudes, and practices of food safety in Saudi dietetics students before and after an educational intervention in a course on food safety and hygiene. We aimed to provide insights into the effectiveness of food-safety education for dietetics students, which could be used to improve dietitian's education and long-term healthcare.

## Methods:

### Study design:

This was a repeated-measure pre/post-intervention study conducted in two consecutive academic years (7-1-2019 to 22-4-2019 and 20-1-2020 to 27-4-2020). The study protocol was approved by the Biomedical Ethics Research Committee (No. 453-21) at King Abdulaziz University. Written informed consent was obtained from all participants.

### Participants:

The participants in this study were female second-year undergraduate students in clinical nutrition (n = 63) enrolled on a course on food safety and hygiene at King Abdulaziz University. Students were informed about the purpose of our study and asked to complete a questionnaire on two occasions: before the intervention (before the first lesson of the course) and after the intervention (after the last lesson of the course).

### Study questionnaire:

The questionnaire was adapted from a validated study [19] with some modifications to suit the target population and to cover key food-safety topics from the course content. Content and face validation of the questionnaire was done by experts in food science and microbiology. Reliability was also tested using a pilot study and Cronbach's alpha coefficient. Good reproducibility for the questionnaire was obtained were Cronbach's alpha coefficient = 0.734 [20]. The pilot study was conducted with 20 undergraduate dietetics students to evaluate the clarity, suitability of wording, and the mean time needed for completion of the questionnaire. Based on these results, some modifications were made. The results of the pilot test are not included here. The questionnaire was created on a Google™ form and was divided into four sections. The questionnaire comprised 49 questions, and it took ∼15 min to complete the questionnaire. The first section of the questionnaire was designed to determine the demographic characteristics of students (four questions). These included questions on age, marital status, having/not having children, and whether children helped to prepare food. The second section was designed to assess knowledge of food safety and consisted of 24 questions. Three sub sections covered are food poisoning (12 questions), food handling (five questions), and personal hygiene (seven questions). The choices for answers were "yes" "no", or "I do not know". Items were scored as a correct response (1 point) and incorrect response (0 points). The third section was created to assess attitudes toward food safety and comprised 10 questions. The choices of answers were "yes", "no", or "I do not know". Items were scored as a correct response (1 point) and incorrect response (0 points). The fourth section was designed to assess the practice of food safety and consisted of 21 questions. Four subsections covered purchasing and storage of food (six questions), preparation and cooking of food (six questions), utensils and equipment used for food preparation (three questions), and personal hygiene (six questions). The choices of answers were a combination of "always" "sometimes", or "never", and closed-end questions of multiple-choice. Items were scored as best practice (2 points) and incorrect practice (0 points).

### Statistical analysis:

Descriptive statistics were applied in the form of frequencies and percentages for categorical data, mean and standard deviation for quantitative data. The McNemar test was applied for nominal data to find a change in proportion for paired data. The paired t-test was applied to test for the difference in the mean value of a continuous variable before and after the educational intervention. Pearson’s correlation coefficient was employed to test the scores for the knowledge, attitudes, and practices of food safety before and after the course. P < 0.05 was significant. Data were analysed using SPSS 25 (IBM Corporation, Armonk, NY, USA).

## Results:

### Participant characteristics:

The study cohort comprised 63 unmarried female students. Their age ranged between 19 years and 26 years (mean, 19.9±1.4 years). Their participation in preparing food improved after the course in food safety and hygiene, whereby the percentage of students preparing food increased from 79.4% to 85.7%, but this difference was not significant.

### Knowledge of food safety:

With regard to food poisoning, Table 1 shows that before the course 58.7% of students knew that microorganisms may not be destroyed in a refrigerator or freezer. However, this percentage increased to 96.8% after the course. Also, before the course, 39.7% of students could recognize that keeping a prepared salad at room temperature for >2 h may result in food poisoning, and this percentage increased to 74.6% after the course. The percentage of students who could recognize that thawing frozen food at room temperature and refreezing frozen food after thawing may cause food poisoning increased from 25.4% and 61.9% before the course to 57.1% and 66.7% after the course, respectively. The percentage of students who knew that inadequately reheated food leftovers and inadequately boiled raw milk may cause food poisoning increased from 42.9% before the course to 79.4% and 65.1% after it, respectively. The percentage of students who could recognize that food contaminated with poisoning bacteria does not always look and taste abnormal increased as a result of the course from 63.5% to 74.6%. Overall, the knowledge of students regarding food poisoning improved significantly (p < 0.001) after the course (7.76±1.71 versus 9.9±1.43). With respect to food handling, before the course 73%, 69.8%, 52.4%, 82.5%, and 49.2% of students knew that it is safer to cook food quantities for ≤1 day, prepared food should not be kept >2 h outside a refrigerator, food purchased first should be consumed first, hot food should not be stored immediately in a refrigerator, and that raw meat should be stored on the lowest shelf of a refrigerator, respectively. However, after the course, these percentages increased to 92.1%, 93.7%, 73%, 85%, and 98.4%, respectively. Overall, the knowledge of students regarding food handling improved significantly (p < 0.001) after the course (3.27±1.05 versus 4.43±0.71). Knowledge of the students that people presenting with diarrhea, vomiting, influenza, or sore throat should not prepare food increased from 63.5% before the course to 100% after it. Also, knowledge that nails on hands should be unvarnished to prepare food safely increased from 49.2% before the course to 63.5% after the course. The percentage of students who knew that apparently healthy people may contaminate food with food-poisoning microorganisms increased from 54% before the course to 84.1% after it (Table 1 - see PDF). Overall, the knowledge of students regarding personal hygiene improved significantly (p < 0.001) after the course (5.48±1.06 versus 6.27±0.90). The total score for knowledge increased as a result of the course from 16.51±2.60 to 20.60±2.01 (p < 0.001).

### Attitudes regarding food safety:

Improvement in the response of students to all attitude-based statements towards food safety was observed except for the statement of "I am willing to obtain more food safety knowledge" because the response remained identical after the course (Table 2 - see PDF). The greatest improvements were observed concerning the statements of "Improper storage of foods may cause food poisoning" and "It is necessary to check the temperature settings of chillers and freezers regularly to reduce the risk of food poisoning" because the percentage of students agreeing increased from 84.1% before the course to 98.4% after it. The total score for attitudes towards food safety increased as a result of the course from 9.16±1.43 to 9.92±0.27 (p < 0.001).

### Self-reported practices regarding food safety:

All items of purchasing and storage practices improved after the course (Table 3 - see PDF). The greatest improvement was observed regarding the practice of storing raw meat or poultry on the bottom shelf of a refrigerator (33.3% to 74.6%). Comparing answers before and after the course, the practice of consuming food purchased first increased from 41.3% to 58.7%, not storing cooked food while still hot in a refrigerator increased from 54% to 71.4%, and storing cleaning chemicals separately from foods and utensils increased from 79.4% to 96.8%. Overall, the practices of purchasing and storage of food improved significantly (p < 0.001) after the course (3.51±1.26 versus 4.65±1.23). Improvements in practices were observed with regard to all items of the preparation and cooking of food after the course (Table 3 - see PDF). The greatest improvement was observed with regard to thawing frozen food of animal origin in a refrigerator (22.2% before the course and 58.7% after the course), followed by the practice of never leaving cooked food on a kitchen counter for >2 h (from 15.9% to 47.6%) and the practice of never refreezing thawed frozen food (28.6% to 60.3%). Overall, the practices of the preparation and cooking of food improved significantly (p < 0.001) after the course (2.0±1.19 versus 3.46±1.70). A slight improvement was observed with regard to the practice of cleaning the utensils and equipment used for food preparation with water and detergent (from 82.5% before the course to 87.3% after it) and always drying utensils and equipment (from 58.7% before the course to 69.8% after it). Overall, the practice of cleaning the utensils and equipment used for food preparation improved slightly from 1.84±0.88 before the course to 1.87±0.79 after the course, but this difference was not significant. Improvements were observed regarding the practices of never preparing food if suffering from diarrhoea, vomiting, influenza, or a sore throat (from 33.3% before the course to 69.8% after it), always washing hands before food preparation (from 77.8% before the course to 90.5% after it), always washing hands using warm water and soap (from 76.2% before the course to 92.1% after it), and always rubbing fingertips between fingers and the wrist during hand washing (from 42.9% before the course to 76.2% after it) (Table 3 - see PDF). Overall, the practices of personal hygiene improved significantly (p < 0.001) after the course (3.65±1.11 versus 4.79±1.12). The total score for practices increased as a result of the course from 11.0±3.10 to 14.78±3.41 (p < 0.001).

### Correlation between the scores for knowledge, attitudes, and practices before and after attending the course on food safety and hygiene:

A significant positive correlation between the scores for the knowledge and practices of food safety was observed before the course (Pearson's correlation coefficient (r) = 0.449, p < 0.001). A significant positive correlation was observed between scores for the knowledge and attitudes towards food safety before the course (r = 0.473, p < 0.001). However, there was no significant correlation between the scores for the knowledge and attitudes of food safety (r = 0.059, p = 0.644) or scores for the knowledge and practices of food safety (r = 0.081, p = 0.528) after the course.

## Discussion:

This interventional study aimed to evaluate the knowledge, attitudes, and practices of food safety of undergraduate dietetics students before and after attending a course on food safety and hygiene. It was demonstrated in this study that the overall knowledge, attitudes, and practices of food safety of dietetics students improved significantly after the course (p < 0.001). This finding supports the result of a similar study conducted by Yarrow et al. [21] on university students. They revealed that an educational intervention on food safety improved the attitudes, beliefs, knowledge, and self-reported practices of students. In addition, a study in Ukraine assessed the effect of a comprehensive curriculum on food safety on students, faculty, and staff, as well as industry and governmental employees. They reported that knowledge, attitudes, behaviours, and skills regarding food safety improved significantly after delivery of the curriculum, and that these factors were maintained 6 months after training had finished [22]. In the present study, the course had a significant impact on the food-safety knowledge of students because the scores for overall knowledge increased from 16.51±2.60 before the course to 20.60±2.01 after the course (p < 0.001). This finding is consistent with data from the results of a study by Asmahan et al. showing that a food-safety training program was the key factor in increasing knowledge among female students at Qassim University (Buraydah, Saudi Arabia) [23]. Other scholars have investigated the influence of a university curriculum on the level of food-safety knowledge and found that students from food-/health-related faculties had a significantly higher score for food-safety knowledge compared with students from other faculties [17, 24 and 25]. Those findings are in line with data from a study by Yusof and co-workers [15], who reported that dietetics students scored higher for food-safety knowledge than food handlers because they had studied about food safety. Before the course, 96.9% of students knew that microbial growth was faster at room temperature than in a refrigerator. However, students' knowledge regarding keeping prepared perishable food at room temperature for >2 h and thawing frozen food at room temperature before the course was only 39.7% and 25.4%, respectively. These data are consistent with results from studies conducted with university students from Greece, Lebanon, and China, where the percentage of correct responses for thawing frozen food was low [24, 26 & 27]. However, in the present study, after the course, improvement in the knowledge of students regarding these concepts was observed, which is likely to reduce the risk of foodborne illnesses. Moreover, students' knowledge regarding the handling of leftover food before the course was inadequate but improved significantly after the course. This phenomenon was also reported by Evans and colleagues in 2021 [28]. They investigated the awareness and attitudes of student dietitians towards food safety from three international institutions. They found that understanding of the safe handling of food leftovers was the lowest among dietetics students from all institutions; only 46% described appropriate reheating practices. We observed that, after the course, the food-handling knowledge of students was improved with regard to consumption of food purchased first as well as storage of prepared food and raw meat in a refrigerator. Preventing long-term storage of food products is important, especially among vulnerable patient groups. Furthermore, significant improvement in the knowledge of personal hygiene of students was observed after the course, which is essential in preventing contamination of food and foodborne illnesses. Most students had a positive attitude before the course, but it improved significantly after the course. Similar findings were reported by Yellow et al. in 2009: an educational intervention improved the attitude of university students. Also, an interventional study showed that teaching the content of food-safety guidelines proposed by World Health Organization positively changed the attitudes of participants towards food safety [29]. Furthermore, attitudes toward food safety and the behaviour of college students and agribusiness students improved significantly as a result of a Food Safety System Management curriculum offered to students in Yerevan (Armenia) [30]. Students were in total agreement that food safety was important to their future as dietetics professionals. In addition, students showed that they would like to increase their knowledge of food safety and hygiene, and were willing to change incorrect food-handling practices. Those data are consistent with results from a study conducted in China in which >80% of college students were willing to improve their knowledge of food safety and to change their inappropriate food-safety practices [27]. In the present study, after the course, students showed they were in absolute agreement with regard to personal hygiene and avoiding cross-contamination to prevent foodborne illnesses. Similar improvements in the attitudes of agribusiness university students have been reported with regard to cross-contamination, Good Manufacturing Practices and personal hygiene by the end of a program in food-safety training [30]. Dietetics professionals, as food-safety educators, should concentrate mainly on personal hygiene, adequate cooking, and avoiding cross-contamination because they are considered the most important factors for reducing foodborne illnesses [31]. Overall, the food-safety practices of students in the present study improved, which showed that students understood that foodborne illnesses are preventable if they apply the concepts of food safety and hygiene in all settings. Similarly, one study found that university students in health sciences with greater knowledge of food hygiene had better reported practices [32]. The response of students improved after the course with regard to several concepts of appropriate purchasing and storage practices, including reading the expiry date and storage of several food items. Moreover, food preparation and cooking practices were improved significantly after the course, including thawing and refreezing food, leaving food on a kitchen counter for >2 h, and dealing with food leftovers. Overall, these practices are important to prevent food contamination and foodborne illnesses. A World Health Organization report [33] showed that 45.6% of outbreaks of foodborne illness were due to temperature abuse during food processing, poor refrigeration, and inappropriate storage temperatures of leftover food or recently cooked meals. With respect to personal hygiene, the hand washing practice of students improved significantly after demonstration of the appropriate hand washing method in the course. Similar improvement in students' hand washing skills after delivery of food-safety training has been reported in Armenia [22]. We revealed a significant positive correlation between knowledge and practices or knowledge and attitudes for food safety among students before the course. Before the course, the students' knowledge derived from their personal experiences was related to their attitudes and practices when they answered the questionnaire, which was reflected in the correlation obtained. A significant correlation was found between food-safety knowledge and food-handling practices (r = 0.406, p < 0.001) and food-safety attitudes (r = 0.651, p < 0.001) among university students in Turkey [34]. Nevertheless, another study reported a negative correlation between total knowledge, attitudes, or practices among dietetics students [15]. Also, a relatively positive correlation was found between knowledge and practices or knowledge and attitudes of food safety among students after the course, but this was not significant and could be attributed to the small sample size. This finding indicates that knowledge leads to positive practices and attitudes. It also indicates that despite the good food-safety knowledge students obtained after the course, they did not implement this knowledge fully into practices and attitudes. This phenomenon emphasizes the need for interactive activities and practical sessions in the course so that students can apply their practical knowledge in real-life activities and obtain a deeper understanding of the topics in the course. Similarly, a study conducted in Malaysia reported that although students have good food-safety knowledge, this did not translate into safe food-handling practices [25]. A study in Turkey showed that food-safety courses in the curriculum led to an increase in the level of food-safety knowledge of veterinary-medicine students, which positively influenced attitudes on food safety. However, students had problems putting their knowledge and attitudes into practice [35]. This is the first study conducted in Saudi Arabia to evaluate the effectiveness of an educational intervention on food safety among dietetics students. The significant improvement in the knowledge, attitudes, and practices of students shown after the course highlights the importance of inclusion of food-safety education in dietetics programs. Doing so would enable dietetics students to provide appropriate food-safety advice to high-risk patients and the broader community. In addition, such courses could help in preparing dietetics professionals to apply for a career in the food industry, where they could provide important guidance and influence the development and marketing of food products.

## Limitations of the study:

Our study had three main limitations. First, the questionnaire was measuring key aspects of the course content, whereas in the future it could be designed to measure broader content (e.g., international regulation of food safety, Hazard Analysis Critical Control Point, and foodborne pathogens). Second, the study was self-reporting, which might have led to biases because respondents may not have self-reflected their actions while answering. Third, our findings relate to undergraduate dietetics students in Saudi Arabia: students from other countries could respond differently.

## Conclusions:

The course on food safety and hygiene helped to improve the knowledge, attitudes, and self-reported practices of food safety by dietetics students. Educators should consider an effective plan for education of food safety for dietetics students to promote deeper understanding of the food-safety needs of the public (especially vulnerable patient groups). Future research could include students' observations before and after a course to discover if changes in attitudes and practices translate into appropriate food-safety practices in real-world conditions. This study was completed before the coronavirus 2019 pandemic, and future research is required to discover the impact of this pandemic on the knowledge, attitudes, and practices of food safety and hygiene.
